# A lightweight hybrid framework integrating convolutional neural networks and fast Fourier transform for reliable and calibrated ECG-based cardiac abnormality detection

**DOI:** 10.1371/journal.pone.0354834

**Published:** 2026-07-29

**Authors:** Abdoul Malik, Selim Aras

**Affiliations:** 1 Department of Intelligent Systems Engineering, Graduate Education Institute, Ondokuz Mayıs University, Samsun, Türkiye; 2 Department of Electrical and Electronics Engineering, Engineering Faculty, Ondokuz Mayıs University, Samsun, Türkiye; Universiti Tunku Abdul Rahman, MALAYSIA

## Abstract

The electrocardiogram (ECG) is an essential non-invasive tool for detecting cardiac abnormalities; however, accurate interpretation often requires specialized expertise that may be unavailable in resource-limited clinical settings. While deep learning models have demonstrated high classification performance, many existing architectures remain computationally intensive and lack assessments of predictive reliability, hindering their deployment in clinical decision support systems. In this study, we propose a lightweight hybrid framework integrating Convolutional Neural Networks (CNN) and Fast Fourier Transform (FFT) components. This architecture combines time-domain morphological representations learned from raw ECG signals with physiologically relevant spectral features to enable accurate and efficient classification. Unlike previous approaches, this work emphasizes reliable model evaluation by incorporating probability calibration and a rigorous patient-wise validation protocol. The proposed method was evaluated on the publicly available PTB-XL dataset using the official 10-fold cross-validation protocol for both binary and five-class multi-label classification. In addition to conventional discrimination metrics, model reliability was assessed using Expected Calibration Error (ECE). The model achieved an accuracy of 92.42% and an AUC of 97.8% for binary classification, alongside a macro-AUC of 92.46% for five- class multi-label classification. Calibration analysis demonstrated well-calibrated probability estimates with low ECE values. Despite its competitive performance, the architecture is highly efficient, containing only 87K parameters and 0.26 GFLOPs. These findings highlight the potential of lightweight hybrid architectures combined with calibration-aware evaluation to support reliable AI-assisted ECG diagnostics in resource-constrained healthcare settings.

## Introduction

Cardiovascular diseases (CVD) account for approximately 32% of global mortality and represent one of the most significant public health issues worldwide [1]. Early diagnosis plays a critical role in reducing morbidity and mortality associated with cardiac disorders [2]. The electrocardiogram (ECG) is one of the most widely used non-invasive diagnostic tools for evaluating the electrical activity of the heart [3]. However, accurate ECG interpretation requires substantial clinical expertise, which is often unavailable in low- and middle-income countries due to the shortage of trained cardiologists [4]. This limitation frequently leads to delayed diagnosis and treatment, particularly in rural and resource-constrained healthcare systems. Consequently, the development of reliable automated ECG analysis systems has become increasingly important to support clinical decision making and improve access to early cardiac screening. Many studies have looked into using machine learning algorithms to automate diagnosis using ECG signals in order to overcome the limitations of manual diagnosis.

Novel methods for automated ECG-based diagnosis have been made possible by recent advances in deep learning [5] and have significant potential to support early diagnosis and triage processes. Various architectures, including convolutional neural networks (CNN), recurrent neural networks (RNN), Transformers, and hybrid models, have demonstrated promising performance [6] and in some cases achieved results comparable to expert cardiologists [7,8]. However, the generalizability and reliability of these models remain subjects of ongoing discussion. Many studies rely on private datasets, which limits reproducibility, carry the risk of patient-dependent data leakage, and do not evaluate clinically critical metrics such as model calibration [9,10].

Among these studies, Strodthoff et al. [11] evaluated the performance of various CNN architectures on the PTB-XL dataset for ECG classification. This study focuses on anomalies such as myocardial infarction and cardiomyopathy. CNNs operating directly on raw signals achieved high accuracy, recall, and AUC levels. These results provide a valuable benchmark for future research in automatic ECG analysis. Wang et al. [12] proposed a CNN integrated with a non-local convolutional block attention module (NCBAM) in their study. Their model improves the capture of local and global relationships in ECG feature maps. Tested on the PTB-XL and MIT-BIH datasets, the model demonstrated its effectiveness in identifying cardiac arrhythmias, achieving 0.9314 AUC and 0.8507 Fmax values. Safdar et al. [13] combine a data augmentation technique based on ECG segmentation and reorganisation with a four-layer CNN model to efficiently manage imbalanced data. With five-fold cross-validation, their model achieved 89.87% accuracy, 88.99% recall, and 0.291 loss, emphasising its effectiveness for generalised classification. Haque and Akhtar [14] investigated ensemble models to classify ECG signals into five top-level classes (NORM, MI, STTC, CD, HYP) and 23 subcategories. Architectures such as VGG16 and AlexNet were tested on PTB-XL. While the VGG16-based model achieved 87.10% accuracy and 0.9448 AUC, the ensemble model reached 86.90% accuracy with 0.9431 AUC.

Martin et al. [15] proposed a Deep Long Short-Term Memory (Deep-LSTM) network for detecting myocardial infarction (MI) in real-time using Lead II ECG signals. Their model was trained on PTB-XL dataset and validated on the PTB dataset, achieving an overall accuracy of 84.17%, sensitivity of 78.37%, and specificity of 87.55%. One of the notable quality of their architecture was its consistent performance across a wide range of sampling frequencies from 202 Hz up to 2.8 kHz, suitable for real-time monitoring. In a separate investigation, Vasconcelos et al. [16] conducted a comparative analysis between CNN based on time-domain (TD) and frequency-domain (FD) representations for atrial fibrillation (AFIB) detection. The used PTB-XL, LUDB, and KURIAS datasets. The FD model achieved an F1 score above 0.81 with transfer learning and the TD model achieved F1 scores below 0.53. their findings highlighting the superiority of frequency-domain approaches for cross-dataset generalization. Yang et al. [17] designed MVMS-Net, a neural network architecture that combines multiple views and scales for multi-label classification. It allow capturing features at various levels to process complex ECG signals. wich demonstrated higher accuracy in multi-label classification capabilities. Yang et al. [18] used Siamese networks to improve anomaly detection in ECG signals while reducing the need for manual annotation. The model demonstrated its effectiveness, particularly in data-constrained scenarios with an AUC value of 0.90%. Zhu et al. [19] proposed a CNN-based model combined with feature selection RFE. Their model achieved an F1 score of 0.902 and a recall of 0.889 by leveraging CNNs for feature extraction and improving the selected features with RFE.Thier finding showed the effectiveness of combining deep learning with feature selection techniques in ECG classification. Śmigiel et al. [20] presented deep learning model, including CNN and SincNet coupling with entropy-based features for ECG signal classification. The CNN combined with entropy-based features achieved the highest accuracy of 0.892 highlighting the potential value of entropy measurements in enhancing the performance of deep learning models for ECG classification.

In another study, Śmigiel et al. [21] extracted entropy features from raw ECG signals and QRS complexes. Then using Deep Neural Networks (DNNs) for ECG classification. Their model achieved an accuracy of 90.2%. Demonstrated the effectiveness of extracting fine-grained signal characteristics to enhance classification performance. In a separate investigation, Pałczyński et al. [22] examined a range of machine learning algorithms including XGBoost, RF, KNN, DT, SVM and Few-Shot Learning (FSL) by applying different kernel configurations. They used R-wave detection and QRS extraction as feature extraction techniques to classify patients as healthy or unhealthy. The FSL network demonstrated a superior accuracy range of 93.2% to 89.2%, proving its effectiveness, especially in scenarios with limited data availability. Similarly, Elyamani et al. [23] proposed a deep residual 2D CNN for the classification of cardiovascular diseases in their study. Although their study did not use specific feature extraction methods, their model achieved an accuracy of 87.85%. Sharma and Eskicioglu [24] deployed a 1D CNN for ECG classification targeting real-time applications using a TensorFlowLite model on Raspberry Pi hardware. They trained their model on 12-lead ECG signals and achieved an accuracy rate of 81.21%. By the way, prove its applicability for lightweight and portable ECG diagnostic systems. Overall, the literature indicates a tendency toward increasingly sophisticated models. However, there is still a clear possibility to systematically combine the strengths of deep learning with physiologically relevant handcrafted characteristics.

Recent studies have increasingly demonstrated the efficacy of Fourier decomposition methods for frequency-domain feature extraction in biomedical signal processing, frequently pairing these techniques with machine learning and deep learning models to improve classification robustness. For instance, Li and Chen [25] proposed an innovative deep feature learning framework where the Fast Fourier Transform (FFT) is used to convert 1D EEG signals into a 2D frequency matrix. Allowing high-level features to be extracted using a PCANet and classified via a Support Vector Machine (SVM) to detect epileptic seizures. Similarly, Gao et al. [26] utilized FFT to extract frequency features (such as the gamma band) from noise-reduced EEG signals, which were subsequently fed into a Pattern Recognition Network to diagnose epilepsy with high accuracy. For cardiac monitoring, Fatimah et al. [27] employed the Fourier Decomposition Method (FDM) to decompose ECG signals into narrow-band Fourier intrinsic band functions (FIBFs). By extracting statistical features from these FIBFs and applying an SVM classifier, they achieved highly accurate inter-patient arrhythmia detection. Furthermore, Lekkas et al. [28] systematically evaluated various 1D-to-2D transformation techniques for ECG and EEG classification using Convolutional Neural Networks (CNNs). They highlighted that the Short-Time Fourier Transform (STFT), which captures dynamic time-frequency representations, allows advanced CNN models (like LeNet-5) to achieve high accuracy. Expanding into multi-modal physiological analysis, Xiang et al. [29] integrated FFT-derived frequency-domain features with traditional time-domain features from wearable sensors (electrodermal activity, heart rate, and skin temperature). Their dual-stream CNN architecture processes the time and frequency domains independently before feature fusion, significantly improving real-world stress detection. While these contemporary methods often rely on 1D-to-2D spectral transformations or multi-stage decompositions that significantly increase computational complexity and parameter overhead, they highlight a distinct gap that our proposed framework addresses: integrating explicit, low-dimensional 1D FFT spectral features with a streamlined 1D CNN to preserve both edge-device efficiency and clinical transparency without sacrificing diagnostic performance.

The PTB-XL dataset [30] has become an important reference for deep learning-based ECG analysis studies due to its 12-lead, large-scale, and multi-label structure. CNN-based models [11], multi-scale convolution approaches [13] and GRU and Transformer-based hybrid structures [17, 31] have reported high accuracy rates on this dataset. Nevertheless, several methodological limitations remain. The majority of these studies do not take into account the multi-labeled nature of ECG signals, which reflects clinical reality accurately and is a fundamental aspect of suitable modeling. Recently, several high-performance results reported by various studies have been shown to be more optimistic than they actually deserve due to random data splitting (80–10–10) and patient-dependent sample mixing [11,20]. This highlights the need for accurate data partitioning, and measurable calibration, in addition to high performance, for clinical applications. Furthermore, while a significant portion of the existing literature primarily focuses on discrimination metrics such as accuracy, F1-score, or AUC, while rarely evaluating the reliability of predicted probabilities. From a clinical perspective, reliable probability estimates are essential for safe deployment in decision support systems. Unreliable probability estimates may limit the safe deployment of AI models in clinical decision support systems and is considered a mandatory evaluation step for integration into clinical decision support systems [10,32]. Therefore, we explicitly evaluate model calibration using Expected Calibration Error.

These shortcomings emerge as important factors limiting the direct transferability of high-performance models to clinical decision support systems. Although CNNs have shown remarkable effectiveness when applied directly to raw signals, they frequently lack interpretability with reference to the particular signal factors that influence their decision-making process. On the other hand, the importance of specific ECG characteristics has been demonstrated using an entropy-based feature technique [20]. However, it might not completely extract the temporal complexity of the signals. The integration of traditional signal processing techniques to extract features from the frequency domain is therefore a promising strategy for improving both the interpretability of models and diagnostic performance.

This study addresses the aforementioned gaps by developing a lightweight framework that integrates a CNN and a Fast Fourier Transform (FFT) for enhanced feature extraction, with calibration-aware evaluation. Unlike numerous prior studies that focus primarily on improving classification accuracy, our work simultaneously considers three practical aspects required for clinical deployment: computational efficiency, integration of physiologically meaningful features, and reliability of predicted probabilities. To this end, the proposed model is evaluated on the PTB-XL dataset using the official patient-wise evaluation protocol, and prediction reliability is assessed using calibration analysis.

The aims of this study is to develop a reliable and computationally efficient framework for ECG classification while addressing several methodological limitations frequently observed in existing studies. The proposed approach is evaluated on the publicly available PTB-XL ECG Dataset using a rigorous patient-wise protocol. The main contributions of this work are summarized as follows:

- Lightweight hybrid architecture: We propose a computationally efficient CNN–FFT hybrid architecture containing approximately 87k parameters and 0.26 GFLOPs, demonstrating that competitive ECG classification performance can be achieved with significantly reduced computational cost, making the approach suitable for real-time and resource-constrained environments.
- Patient-wise evaluation protocol: We adopt the official patient-wise evaluation strategy to prevent optimistic bias caused by patient-dependent data leakage, highlighting the importance of rigorous data partitioning for reliable performance estimation.
- Calibration-aware evaluation: In addition to conventional discrimination metrics, we perform a detailed calibration analysis using Expected Calibration Error (ECE) and reliability diagrams, emphasizing the importance of reliable probability estimates for clinical decision support systems.
- Systematic spectral feature analysis: Through an extensive ablation study, we analyze the impact of different spectral feature extraction techniques and show that simple physiologically meaningful frequency-domain representations derived from FFT provide stable improvements compared with more complex multi-transformation approaches.

First, we focus on binary classification in order to distinguish healthy subjects from subjects with cardiac abnormalities. Then, a multi-label classification task is performed to identify five cardiac conditions. The proposed solutions are designed to adapt to the inherent variability of ECG signals, thereby improving diagnostic accuracy and facilitating their integration into clinical practice. The rest of this paper is organized as follows. The Materials and Methods section describes our methodology. The Results section analyzes the experimental results, and the Discussion section examines their clinical implications. Finally, the Conclusion summarizes the main findings and provides recommendations for future research.

## Materials and methods

This study is based on a hybrid deep learning architecture that utilizes both the time and frequency-domain characteristics of 12-lead ECG signals from the PTB-XL dataset. The method consists of the following stages: preparation of the dataset, preprocessing of the signals, extraction of time–frequency features, and training of the hybrid CNN–FFT model. The experiments strictly follow the official PTB-XL 10-fold stratified cross-validation protocol [30], which guarantees patient-wise separation and class balance across folds. Folds 1–8 were used for training, fold 9 for validation and fold 10 for testing. The simultaneous presence of records belonging to the same patient in the training and test sets was strictly prevented; thus, the patient-dependent data leakage problem that can arise in many studies using random partitioning was eliminated. This strategy ensures a more accurate assessment of the model’s performance on individuals it has never encountered before in the real world. We adhered to the TRIPOD+AI (Transparent Reporting of a multivariable prediction model for Individual Prognosis or Diagnosis + Artificial Intelligence) statement guidelines for reporting the development and evaluation of our model (see S1 Checklist).

### Datasets

We used the PTB-XL [30], a publicly available 12-lead ECG database comprising recordings from diverse demographic and clinical settings and contains 21,799 ECG recordings from 18,869 patients, representing a wide range of ages, genders, and clinical conditions. We accessed the dataset via the PhysioNet repository in November 2025. All data were fully anonymized prior to public release, and we had no access to personally identifiable information. The data were originally collected with appropriate ethical approval and informed consent, as described in the original publication. This study is a secondary analysis of publicly available, anonymized data and did not require additional ethical approval. One of the main features of the PTB-XL dataset is its comprehensive coverage of different heart diseases. Containing a wide range of labeled ECG data, PTB-XL contains high-quality signals in WFDB format (500 Hz and 100 Hz, 16 bits). Clinical utility is ensured by the full metadata and expert annotations given by cardiologists. The dataset is categorized into five principal classes: Normal (NORM), Myocardial Infarction (MI), ST/T Segment Changes (STTC), Conduction Disorders (CD), and Hypertrophy (HYP).

The first approach is a binary classification task designed to distinguish between Normal (healthy) individuals and subjects with cardiac abnormalities (individuals diagnosed with cardiac pathology). Specifically, the Abnormal category includes all ECG records diagnosed with at least one of the four pathological superclasses defined in the PTB-XL dataset (MI, STTC, HYP, and CD). For this approach, ECG recordings were obtained from a 10-second PTB-XL dataset of 17,407 recordings. For the binary classification task, only ECG recordings with 100% diagnostic certainty were included. This methodological choice aims to ensure diagnostic clarity by eliminating ambiguous cases and has been used in several previous studies [19,20]. The distribution of the data for the binary classification is shown in Table 1, which includes 10,384 records for the abnormal class and 7,023 records for the normal class. Normal ECGs were designated 0, and aberrant ECGs were labelled 1. All records were sampled at 500 Hz.

**Table 1:** Distribution of samples across different classes in the dataset.

Classification Task		Super classes	Number of Recordings
**Binary**	**Normal**	NORM	7023
	**Abnormal**	MI	10384
		STTC	
		CD	
		HYP	
**Five Class Multi-label**		NORM	9,514
		MI	5,469
		STTC	5,235
		CD	4,898
		HYP	2,649

In the second task, five superclasses were classified using multi-class classification with multi-label records. The distribution of these samples across the relevant superclasses is displayed in Table 1. Because the dataset is multi-label, the total number of records is less than the sum of the records per class. Each recording was truncated or zero-padded to a fixed length of T = 5000 samples (10 s at 500 Hz). Truncation keeps the first 5000 samples, while shorter recordings are padded with zeros at the end to produce a consistent input tensor of shape (N, 5000, 12).

To evaluate the baseline clinical characteristics of the study cohort, we analyzed the age distribution across the five principal diagnostic superclasses. A non-parametric Kruskal-Wallis test has been used to evaluate age variations among the groups, as the age distribution is not normal. Table 2 summarizes the age distribution across the five PTB-XL dataset diagnostic superclasses. Statistical testing showed highly significant differences in age among the groups (p < 0.001), demonstrating that pathological cohorts, particularly Myocardial Infarction (MI), ST/T Segment Changes (STTC), and Conduction Disorders (CD), naturally exhibit a significantly older age profile compared to normal healthy (NORM) within this multi-label clinical dataset.

**Table 2:** Demographic characteristics of the population by diagnostic class.

Diagnostic Class	Number of samples	Median Age (Years)	Interquartile Range (IQR) (Years)
NORM	9,514	54	40–65
MI	5,469	67	58–76
STTC	5,235	68	58–77
CD	4,898	68	57–77
HYP	2,649	68	58–77

### Data preprocessing

ECG signals directly reflect the heart’s electrical activity, but during the recording process, they are exposed to numerous physiological and environmental noises and artifacts. Baseline wander commonly encountered in clinical applications is mostly caused by respiratory movements, slow changes in electrode-skin impedance, and small movements in body position [3,9]. Electromyographic (EMG) noise associated with muscle activity occurs particularly during involuntary muscle contractions and patient movements, causing high-frequency components to be added to the ECG signal [33]. Additionally, high-frequency interference related to the measurement environment and electronic equipment can make it difficult to reliably analyze QRS morphology and the ST segment.

The literature argue that analyses performed without removing noise such artifacts can negatively affecting model generalizability [34]. Therefore, in our study, each ECG signal underwent a systematic preprocessing step before analysis. A 5th-order Butterworth bandpass filter with cutoff frequencies of 0.5–50 Hz was applied to eliminate baseline drift, muscle noise, and high-frequency interference. The filtering process was performed by processing the signal sequentially in both forward and reverse directions; thus, phase shift was completely eliminated, preserving the morphological integrity of the P–QRS–T complexes [33]. This approach is critical for the reliable representation of phase-sensitive components, such as the ST segment and T wave.

**Fig 1:**
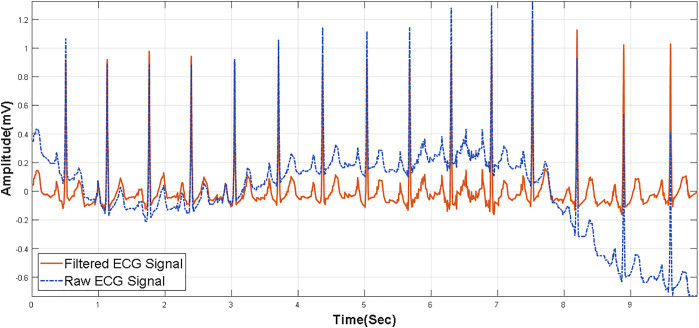
Comparison of a raw and filtered ECG signal.

Following the filtering step, each derivation was subjected to Z-norm normalization and scaled so that each signal had a mean of zero and a standard deviation of one. This process reduces inter-individual amplitude differences and electrode placement-related scale variations, enabling the model to learn a patient-independent and more stable representation. Many deep learning-based ECG studies in the literature report limited preprocessing steps or only specify filter frequency ranges [33]. In contrast, the detailed filtering and normalization strategy used in this study helps ensure that the classification performance is primarily based on physiological signal components. The difference between raw and filtered ECG signals is visually presented in Fig 1 using an sample record.

### Extraction of time and frequency-domain features

In the proposed approach, the aim is to represent both the morphological structures of ECG signals in the time-domain and the rhythmic and spectral patterns in the frequency-domain. While time-domain information is automatically learned by a convolutional neural network (CNN) directly from raw ECG signals, distinctive features belonging to the frequency-domain are explicitly calculated and added to the model. This integrated structure allows the model to use local waveform features and global rhythmic components simultaneously. To obtain frequency-domain representations, a fast Fourier transform (FFT) was applied to each derivation. The following core spectral features were calculated from the amplitude spectrum and power spectral density obtained from the FFT: The dominant frequency represents the component with the highest amplitude in a specific frequency range and is defined as follows:


$$\begin{array}{c} f_{\text{dom}}=\text{arg}\underset{f_{k}\leq \text{4}\text{0}\;\text{Hz}}{\text{max}}|X[k]| \end{array}$$ (1)

Where X[k] represents the FFT coefficients of the ECG signal, and $f_{k}$ represents the corresponding frequency components. This range has been considered because the majority of physiological information in clinical ECG analyses is concentrated in the 0–40 Hz band. Additionally, to get the energy distribution in specific frequency bands, we calculated the band energy as follows:


$$\begin{array}{c} E_{\left[f_{\text{1}},f_{\text{2}}\right]}=\int_{f_{\text{1}}}^{f_{\text{2}}}P_{xx}(f)\;df \end{array}$$ (2)

$P_{xx}(f)$ defined as the power spectral density of the signal. Band energy provides a distinctive criterion, particularly in pathologies with a pronounced rhythmic character, such as ST–T changes and conduction disturbances. To represent the distribution of spectral information in greater detail, second-order distribution measures such as spectral centroid and spectral width were also calculated. These measures provide complementary information about the rhythmic structure of the signal by defining the center of gravity and spread of spectral energy on the frequency axis. In addition to FFT-based features, time–frequency representations were also obtained using Welch-based power spectral density (Welch-PSD) and discrete wavelet transform (DWT) within the scope of the ablation study. These additional transformations were used to comparatively evaluate the diagnostic contribution of the proposed FFT-based approach and to investigate the effect of different spectral representation methods on classification performance. The results are presented in detail in the ablation study section, showing the contributions of these transformations.

### Hybrid CNN–FFT architecture

The main design objective of the proposed hybrid CNN–FFT architecture in this study is to create a simple yet effective framework that clearly and complementarily represents the time-domain morphology and frequency-domain rhythmic structure in ECG signals. Although deep CNN structures or attention mechanisms perform well, these approaches increase model complexity, computational expense, and clinical interpretability. The proposed architecture intentionally diverges from this trend, adopting a simpler, more explainable, and computationally efficient design.

Time-domain representations are automatically learned by a 1D CNN operating on raw ECG signals. The CNN module consists of three consecutive convolution blocks; each block contains convolution, Batch Normalization, ReLU activation, and MaxPooling components. This structure targets the separation of shape features of the QRS complex, variations in the ST segment, and morphological patterns of the T wave. Instead of being implicitly learned within the CNN, frequency-domain representations are explicitly calculated as physiologically meaningful spectral features and fed into the model. Dominant frequency, band energies, and spectral distribution metrics obtained through the FFT module are transformed into a low-dimensional latent representation. This approach ensures that frequency-domain information is used in a way that is directly interpretable and controllable by the model.

**Fig 2:**
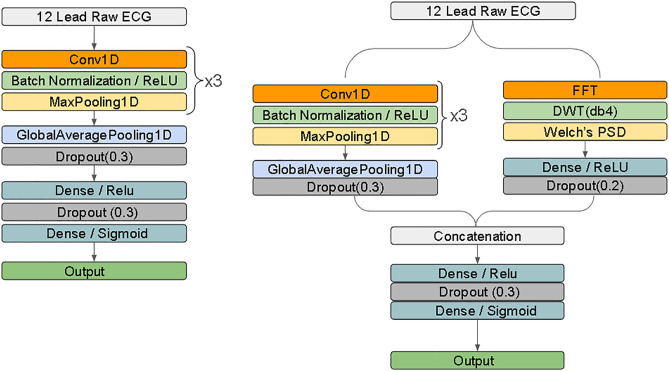
CNN architecture (left) and the proposed hybrid CNN–FFT architecture (right) integrated with FFT based frequency features.

The representations obtained from the CNN and FFT modules were fused in a fusion layer and then class probabilities were generated through fully connected layers. This fusion strategy allows the model to simultaneously evaluate local morphological details and global rhythmic patterns, increasing its discriminative power, particularly in pathologies involving both time and frequency components, such as ST–T changes and conduction abnormalities. The proposed architecture differs from many end-to-end approaches in the literature in that it uses frequency-domain information as an explicit branch rather than embedding it into the CNN. These design choices enable the proposed model to not only deliver high classification performance but also provide practical advantages such as low computational cost, better generalizability, and clinically interpretable decision output. The architectural flow and data interaction between components are presented schematically in Fig 2 and Table 3 provides an overview of the hyperparameter tuning performed in this study.

**Table 3:** Training hyperparameters.

Hyperparameters	Tested Values	Best Values
Number of layers	2–4	3
Number of filters per layer	6, 32, 64, 128, 256	64, 128, 256
Kernel size	3, 5, 7	3
Pool size	2, 3, 4	2
Number of neurons in a dense layer	64, 128, 256	128
Dropout rate	0.2, 0.3, 0.4, 0.5	0.2, 0.3
Optimisation algorithm	Adam, RMSprop, SGD	Adam
Learning rate	1e-2 – 1e-5	1e-3
Loss function	sparse categorical cross-entropy, categorical cross-entropy, binary cross-entropy	sparse categorical cross-entropy, binary cross entropy
Number of epochs	10–50	20, 40
Batch size	16–128	64

### Model training and evaluation metrics

The training of the proposed hybrid CNN–FFT model was performed using the Adam optimization algorithm to balance stable convergence and generalization performance. The learning rate was set to 1 × 10^-3^; dropout (0.3) and L2 norm-based weight regularization were applied together to reduce the risk of overfitting. The loss function was selected as Binary Cross-Entropy for the binary classification scenario and Categorical Cross-Entropy for the five-class classification scenario, in accordance with the problem structure. The number of training epochs was tested between 10 and 50 during hyperparameter optimization. For the binary and five-class classification tasks, 20 and 40 epochs were empirically determined to be the optimal values to guarantee complete loss convergence while effectively preventing overfitting on the training data. This training strategy aims to achieve a structure with more limited complexity but stable learning behavior, unlike the long training times and high-parameter models, this model is intended to generalize consistently not only to the training data but also to patient-independent test data. To generate the confusion matrices, a decision threshold of 0.5 was applied to the output probabilities produced by the sigmoid activation function. In the multi-label setting, each diagnostic category was evaluated independently. All evaluations were performed exclusively on the independent test set (fold 10). When evaluating model performance, the accuracy metric alone was not sufficient; F1 score, ROC–AUC, precision–recall (PR) curves, and confusion matrices were used together to characterize the diagnostic behavior in more detail. It was observed that the F1 score and PR curves gave a more significant performance indicator in comparison to the accuracy value. This was especially true in the case when there was an imbalance between the five classes. One definition of a True Positive (TP) is a positive class that was accurately predicted, while the definition of a True Negative (TN) is a class that was correctly identified as being negative. A false positive (FP) is a mistaken positive forecast for a negative sample, whereas a false negative (FN) is a missing positive example. In addition, to evaluate the statistical reliability of the performance metrics, the 95% confidence intervals (CI) were computed using a non-parametric bootstrap resampling approach. This method involved resampling the independent test set (fold 10) with replacement over 1,000 iterations to generate empirical distributions for each metric, from which the confidence intervals (CI) were estimated.

The classification performance was assessed using standard metrics, calculated according to the following equations.


$$\begin{array}{c} Accuracy=\frac{TP+TN}{TP+FP+TN+FN} \end{array}$$ (3)

$$\begin{array}{c} Recall=\frac{TP}{TP+FN} \end{array}$$ (4)

$$\begin{array}{c} Precision=\frac{TP}{TP+FP} \end{array}$$ (5)

$$\begin{array}{c} F\text{1}Score=\frac{\text{2}*Precision*Recall}{Precision+Recall} \end{array}$$ (6)

In addition, model calibration, which is critical for clinical decision support systems, was also included in the evaluation process. The expected calibration error (ECE) was calculated to quantitatively measure the reliability of the class probabilities produced by the model. This approach reveals not only whether the model predicts the correct class but also how well the predicted probabilities align with the actual occurrence rates. Thanks to this multidimensional evaluation framework, it has been systematically analyzed that the proposed hybrid CNN–FFT model provide high classification performance and produces reliable and interpretable predictions in clinical use. In contrast to many previous studies, this work explicitly incorporates calibration analysis as a primary evaluation criterion, rather than treating it as a secondary metric. This ensures that the model is not only accurate but also reliable for real-world clinical decision-making.

## Results

Within the scope of binary and five-class classification scenarios, the performance of the hybrid CNN–FFT architecture that was developed is presented in this section. In addition, the findings of an ablation study that was carried out to investigate the influence that various feature extraction procedures have on classification performance are provided. The official patient-based 10-fold cross-validation methodology has been followed throughout each and every experiment during its execution. All of the computational analyses and experimental processes were carried out within a framework that was based on Python. By utilizing TensorFlow and its high-level application programming interface (API) Keras, we were able to design, train, and evaluate models. On the other hand, the Scikit-learn library was utilized for the purposes of data processing and the computation of performance measures. This signal and data preparation was accomplished with the help of NumPy and Pandas. SciPy and PyWavelets were utilized in order to carry out the activities concerning the extraction of characteristics.

### Binary classification performance

**Fig 3:**
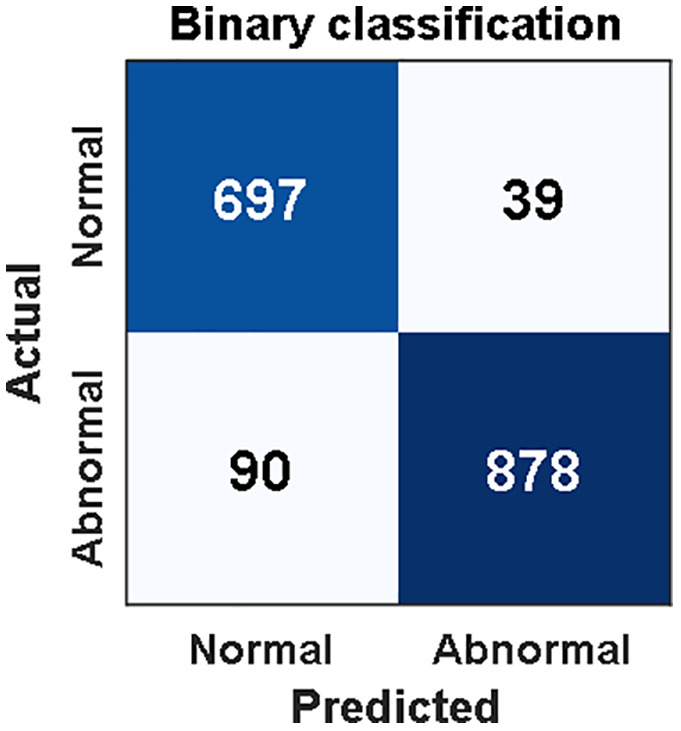
Confusion matrix for the binary classification task.

**Fig 4:**
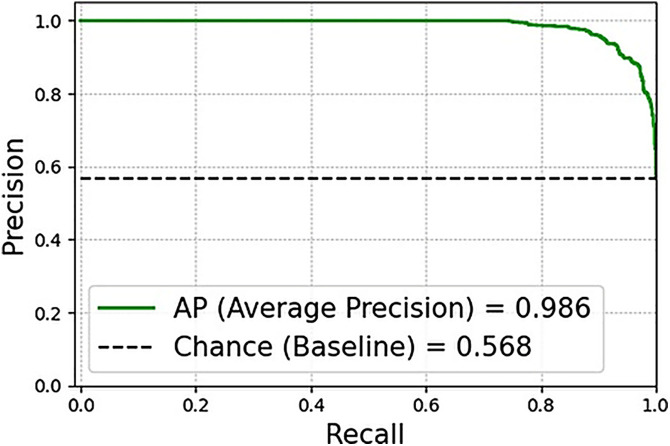
Precision–recall (PR) curve for the proposed binary classification model.

The finding for binary classification are summarized in Table 4. Our hybrid CNN–FFT approach achieved 92.42% accuracy, 93.13% F1 score, and 97.8% AUC values providing a clear improvement over the baseline CNN model. It is particularly evident in the reduction of the false negative rate. This demonstrates that frequency-domain provide powerful complementary information for distinguishing abnormal cardiac patterns. The confusion matrix for the binary classification task is presented in Fig 3. Examination of the matrix reveals that the model accurately identifies normal records; in the case of samples belonging to the abnormal class, records exhibiting pronounced spectral characteristics are correctly classified. The precision-recall (PR) curve given in Fig 4, shows that a balanced relationship between precision and recall is achieved under different decision thresholds. Additionally, the reliability of the class probabilities generated by the model was evaluated through calibration analysis. The calibration curve reveals that the predicted probabilities are in high agreement with the actual. The fact that the ECE value is low is evidence that the model not only provides accurate classification but is also properly calibrated, both of them are essential for clinical decision support systems.

**Table 4:** Performance metrics for the binary classification task on the test set (with 95% CI).

Model	Accuracy	Precision	Recall	F1-score	AUC (%)
**CNN + FFT**	92.42 (91.5–93.2)	95.74 (94.56–96.92)	90.70 (89.28–92.12)	93.13 (91.86–94.40)	97.8 (97.3–98.3)

### Five-class multi-label classification performance

The class-based performance results obtained for the five main diagnostic superclasses (NORM, MI, STTC, CD, and HYP) are presented in Table 5. The model achieved high F1 and AUC values in the NORM, MI, STTC, and CD classes; it demonstrated a strong ability to represent ischemia-related morphological and spectral changes, particularly in the MI class, with an AUC value exceeding 93%.

**Table 5:** Class-based performance of the multi-label five-class classification model on the test set (with 95% CI).

Class	Precision (%)	Recall (%)	F1-Score (%)	AUC (%)	AUPRC (%)
**CD**	82.3 (78.5–85.9)	66.5 (62.4–70.4)	73.6 (70.4–76.8)	90.7 (89.6–91.8)	81.5 (78.2–84.6)
**HYP**	70.6 (63.3–77.5)	43.9 (38.2–50.0)	54.1 (48.4–59.6)	90.2 (89.2–91.4)	65.8 (59.7–71.4)
**MI**	80.2 (76.5–83.9)	68.4 (64.7–72.2)	73.8 (71.0–76.8)	93.2 (92.2–93.4)	84.4 (81.9–87.1)
**NORM**	81.3 (79.2–83.7)	91.8 (90.0–93.5)	86.2 (84.7–87.8)	94.7 (93.6–95.4)	92.6 (91.1–94.1)
**STTC**	77.9 (74.1–81.5)	71.6 (67.4–75.6)	74.6 (71.5–77.5)	93.5 (92.1–94.4)	82.4 (79.3–85.4)
**Overall**	80.0	74.0	77.0	92.46	85.4

**Fig 5:**
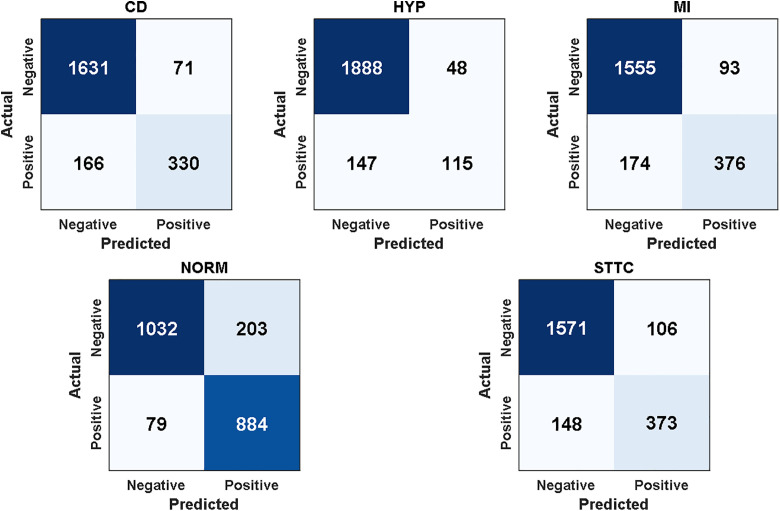
Five-class multi-label classification confusion matrix.

The HYP class has been the most challenging category, as also noted in several previous studies. In this class, despite the relatively low F1 score compared to other classes, the high AUC value indicates that the model can distinguish signals specific to hypertrophy; however, due to overlap between classes, the decision boundaries are more ambiguous. The confusion matrix per class for the five class multi-label classification task is given in Fig 5.

**Fig 6:**
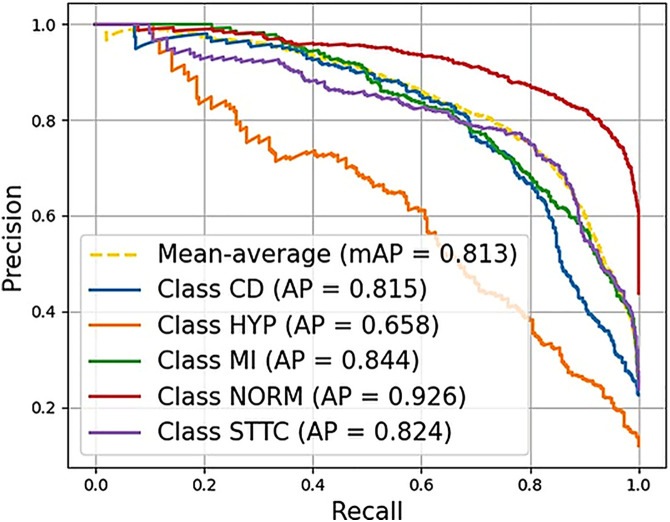
Precision–recall (PR) curves for the proposed five-class classification model.

**Fig 7:**
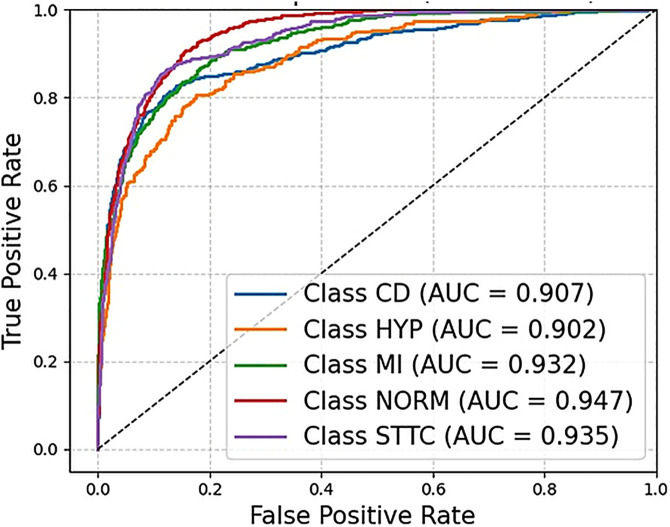
ROC curves for each class in a five-class classification task.

The most prominent class confusion is concentrated in the STTC–HYP and CD–STTC pairs, reflecting overlapping morphological and spectral patterns between these categories. This can be explained by the fact that these classes contain partially overlapping patterns, both morphologically and spectrally. The PR curves and calibration analysis presented in Fig 6 show that the model offers a balanced sensitivity–recall relationship in a multi-class scenario and is well calibrated. The ROC curves showed in Fig 7 reveal high discriminative power in the MI, STTC, and CD classes. When evaluating the overall macro average results, the proposed hybrid CNN–FFT architecture achieved a macro F1 score of 77% and a macro AUC of 92.46%, demonstrating strong overall performance despite the imbalanced class distribution.

### Ablation study

**Fig 8:**
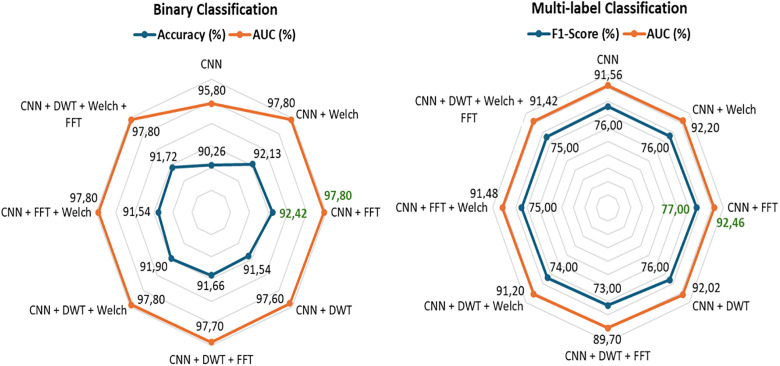
Contribution of FFT-based frequency features in binary and multi-class scenarios.

To evaluate the relative contributions of different feature extraction strategies used, the performance of FFT, Welch-PSD, and DWT-based features were compared. A more intuitive comparison of the numerical results of the ablation are presented in Table 6 and depicted in Fig 8, separately for binary and five-class tasks. While the baseline CNN model achieves 90.26% accuracy and 95.8% AUC in binary classification, the integration of FFT-based features noticeably increased these values. Fig 8 show that FFT-based frequency features provide the most balanced and consistent contribution in both binary and multi-class scenarios.

**Table 6:** Ablation study on CNN-based models for binary and multi-label classification tasks.

Model	Binary Classification		Multi-label Classification	
	Accuracy (%)	AUC (%)	F1-Score (%)	AUC (%)
CNN	90.26	95.8	76	91.56
CNN + Welch	92.13	97.8	76	92.20
**CNN + FFT**	**92.42**	**97.8**	77	**92.46**
CNN + DWT	91.54	97.6	76	92.02
CNN + DWT + FFT	91.66	97.7	73	89.70
CNN + DWT + Welch	91.90	97.8	74	91.20
CNN + FFT + Welch	91.54	97.8	75	91.48
CNN + DWT + Welch + FFT	91.72	97.8	75	91.42

The fact that FFT alone provides a higher contribution compared to Welch-PSD and DWT-based approaches indicates that the discriminative information in ECG signals is largely concentrated in the frequency-domain. However, a performance decline has been observed in variants where multiple spectral transformations are used simultaneously. This suggests that overfitting may result from representational redundancy and increased model complexity. Ablation analysis results clearly demonstrate that using simple physiologically meaningful frequency representations in the proposed hybrid model is more effective than complex and multi-transformation strategies.

The ablation study further highlights the contribution of frequency-domain information to the proposed framework. Among the evaluated configurations, the integration of FFT-derived features yielded the highest overall performance. It is important to note that the Fast Fourier Transform (FFT) itself is a deterministic and non-trainable signal transformation, meaning that it does not introduce additional learnable parameters during feature extraction. Rather than increasing model complexity through additional trainable filters, the FFT branch provides complementary spectral information that is fused with the temporal representations extracted by the CNN. This dual-domain representation enables the model to capture both temporal and frequency-related characteristics of ECG signals, contributing to improved predictive performance on the independent test set. These findings suggest that the observed performance gains are associated with the incorporation of complementary spectral information rather than solely with an increase in model capacity.

### Model calibration analysis

**Fig 9:**
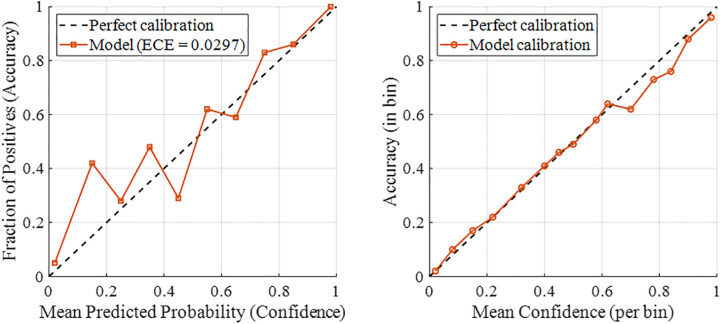
Calibration diagrams of the model for binary (left) and five-class (right) classification tasks.

We performed a calibration analysis to assess the reliability of the model’s decision probabilities and the degree to which the predicted class probabilities align with the actual class. In this context, reliability diagrams were analyzed for both binary and five-class classification tasks and we calculated the Expected Calibration Error (ECE). This assessment is critically important for the reliable use of the model in clinical decision support systems, independent of classification accuracy. Indeed, the literature on clinical prediction models clearly emphasizes that calibration analysis is often neglected and that this constitutes a significant limitation [32]. The visual results of the calibration analysis are presented in Fig 9. The figure shows the fit of the probabilities predicted by the model with the ideal calibration line (45° line) for each confidence interval (bin). In an ideally calibrated model, the predicted probabilities are expected to correspond to the actual positive rates.

The calibration curve obtained in the binary classification task follows a trajectory closely aligned with the ideal line, especially in the 0.3–0.8 probability range. The fact that the calculated ECE value is low (0.0297) indicates that the model reliably ranks decision probabilities. This is critically important in clinical decision support systems to prevent misleading predictions. Generaly, the calibration curves behaved well in the five-class classification situation despite the imbalanced class distribution. The NORM and MI classes had probability estimates near to the ideal line, but the CD and STTC classes had moderate departures. HYP class fluctuations match its data imbalance and morphological heterogeneity. The calibration analysis demonstrated that the CNN–FFT model produces clinically reliable, risk-based probabilistic outputs for decision-making and discriminative classification performance.

## Discussion

### Benchmarking and performance evaluation

We evaluated our hybrid CNN–FFT architecture for both binary and five-class multi- label classification. Our findings allow us to argue that integrating morphological representations learned by CNN in the time-domain with spectral features extracted explicitly in the frequency-domain meaningfully enriched the diagnostic information in ECG signals. In this context, the hybrid approach not only offered higher discriminative power compared to CNN-based models relying solely on the time-domain but also produced reliable probability estimates, which are of critical importance for clinical decision support systems. Precision–recall curves and calibration analyses (Figs 4 and 9) reveal that the model exhibits balanced behavior under different decision thresholds and that the predicted probabilities are consistently aligned with the actual class frequencies.

Unlike many existing studies that primarily focus on maximizing classification accuracy, this study emphasizes reliability and clinical applicability. In particular, calibration analysis is rarely reported in PTB-XL-based studies, despite its critical importance in clinical decision-making. This work underscores the importance of model reliability, emphasizing that calibration should be regarded as a primary evaluation criterion alongside discrimination performance.

The accuracy of 92.42% and AUC value of 97.8% achieved in the binary classification task indicate a clear improvement compared to several studies reported in the literature on the PTB-XL dataset. A comparative analysis presented in Table 7 shows that the proposed approach outperforms the accuracy and F1 score levels reported by [13,19,20]. One of the main reasons for this difference is that the frequency-domain components reduce the false negative rate, especially in abnormal ECG recordings. FFT-based spectral features explicitly represent band energy and dominant frequency changes specific to pathological rhythms, providing the model with discriminative information that morphology-based approaches alone may miss. In addition, the excellent performance of binary classification presents itself as a reliable tool for early diagnosis of heart disease.

**Table 7:** Performance comparison with prior studies on binary classification using the PTB-XL dataset.

Reference	Method Used	Results
[13]	CNN	Accuracy = 89.87%
[17]	Siamese Networks	AUC = 90%
[19]	CNN	F1-score = 90.2%
[20]	Entropy-based features + CNN and SincNet	Accuracy = 89.2%
[21]	Entropy (from QRS complexes) + DNN	Accuracy = 90.2%
[22]	XGBoost, RF, SVM, KNN + Few-Shot Learning + R/QRS features	Accuracy = 89.2%
[23]	2D CNN	Accuracy = 87.85%
[24]	1D CNN	Accuracy = 81.21%
[35]	CNN	Accuracy = 86.58%
**This Study**	**CNN + FFT**	**Accuracy = 92.42%**

The five-class multi-label classification results showed that the hybrid CNN–FFT architecture exhibits strong performance even in a more complex diagnostic problem structure. The high F1 and AUC values obtained in the NORM, MI, STTC, and CD classes reveal that the model can effectively capture both intra-class morphological diversity and inter-class spectral differences. In particular, the AUC value above 93% obtained in the MI class demonstrates that time-frequency representations can effectively capture ischemia-related morphological changes and accompanying spectral changes when used together. The confusion matrix and ROC curves (Figs 5 and 7) reveal that the confusion between the STTC–HYP and CD–STTC classes is more pronounced; this is consistent with the fact that, clinically, these classes contain partially overlapping patterns both morphologically and spectrally.

The HYP class was the most challenging category in this study, as also reported in several previous investigations. Although the F1 score for this class was relatively low compared to other categories, the high AUC value indicates that the model retains a strong capacity to distinguish hypertrophy-specific signaling components when isolated. However, it also indicates that decision boundaries are more ambiguous due to intraclass heterogeneity, class imbalance, and the overlap of hypertrophy patterns with other pathologies. From a clinical perspective, ventricular hypertrophy alters myocardial repolarization and depolarization pathways in ways that directly mimic or co-exist with other cardiovascular abnormalities. For instance, Hypertrophy (HYP) frequently triggers secondary ST-segment shifts and T-wave asymmetries known clinically as a hypertrophic strain pattern, which heavily overlap with ischemic signatures classified under ST/T Segment Changes [36]. Furthermore, the increased muscle mass in hypertrophy prolongs the ventricular activation time. It widens the QRS complex, creating significant morphological and spectral redundancy with Conduction Disorders (CD), such as bundle branch blocks [36,37]. This intrinsic pathophysiological overlap explains why the confusion matrix yields pronounced misclassifications between the STTC–HYP and CD–STTC pairs, representing a classic diagnostic challenge not only for automated deep learning frameworks but also for expert cardiologists in routine clinical practice [36]. This finding suggests that the results of some studies reporting high accuracy in the hypertrophy class may have been partially influenced by methodological factors such as data imbalance or patient-dependent data partitioning.

To situate our finding with the existing study using the PTB-XL dataset, we compared our research with the study delineated in [38], which presents a hybrid Convolutional Neural Network (CNN) architecture and Variational Autoencoder (VAE) techniques for the classification of heart diseases. Their model demonstrated the potential of deep learning for automated ECG interpretation with a high accuracy of 98.51%, specificity of 98.12%, and F1-score of 97.95% for five-class diagnostic tasks. However, their research employed a single-label instead of a multi-label paradigm, in which every ECG is categorized into a single group. Conversely, our methodology specifically addresses multi-label categorization, in which a single ECG recording may contain many cardiovascular diseases. Because realistic cardiac rhythm analysis frequently necessitates identifying many simultaneous abnormalities instead of sole categories, this distinction is clinically meaningful. In our study, we present an improved multi-label ECG classification model that combines CNN and manually extracted features.

As summarized in Table 8, we offer a thorough comparison with seven current and typical multi-label electrocardiogram classification techniques applied to the PTB-XL collection in order to further confirm the efficacy of our suggested approach. They use a variety of architectures, from attention-based and self-supervised learning models to SE-ResNet and multi-branch CNNs. A multi-label classification technique based on SE-ResNet and a k-label sets approach was presented by Yoo et al. [39]. Their method produced one of the best baselines, with an Exact Match of 63.7% and an F1-score of 92.43%. Nevertheless, their approach is not inherently interpretable. Our approach provides integrated interpretability, which improves clinical transparency and reliability, while achieving a comparable Exact Match (63.9) with a moderate F1-score (77%).

**Table 8:** Performance and computational complexity comparison with prior studies on multi-label ECG classification using the PTB-XL dataset.

Reference	Split Ratio	Method	Exact Match	F1 Score	Macro-AUC	Parameters
[39]	60%−20%−20%	SE-ResNet	63.7	92.43	–	–
[17]	80%−10%−10%	MVMS-Net	–	41.48	92.58	0.39 M
[41]	80%−10%−10%	MBSR+SHAF	–	74.65	92.97	–
[42]	10-fold cross-validation	3D-STLNet + MHA	–	80.43	92.5	–
[31]	80%−10%−10%	Leadwise clustering multi-branch network	–	46	93.45	–
[40]	80%−10%−10%	WildECG	–	48	84.5	0.313M
[43]	80%−10%−10%	ECA + SENet + ResNet	62.9	91.70	91.92	11.7 M
**This Study**	**10-fold cross-validation**	**CNN + FFT**	**63.9**	**77**	**92.46**	**0.087 M**

Zhou and Chen [31] developed a leadwise clustering-driven multi-branch architecture that categorizes leads according to clinical significance. Notwithstanding its conceptual innovation and an AUC of 93.45, their model attained an F1 score of only 46%, which is far inferior compared to our method’s score. Avramidis et al. [40] introduced WildECG, a supervised approach designed for multipurpose ECG feature extraction. Although proficient in representational learning, their model attained a 48% F1 score and 84.5% AUC on PTB-XL and was not specifically optimized for the classification of multiple labels. Furthermore, interpretability had not been considered. Zeng et al. [34] suggested an ECA + SENet + ResNet model that integrates global (SENet) and locally (ECA) mechanisms of attention with an advanced residual architecture to concurrently improve description of features and label differentiation. They reached an Exact Match of 63.8%, an F1 score of 91.81%, and an AUC of 91.92%.

Yang et al. [17] Designed a multi-view and various-scale deep neural network system intended for capturing lead-level temporal and spatial data. Despite achieving a macro-AUC of 92.58%, the F1 Score of 41.48 is considerably inadequate. Moreover, their design lacked an interpretability analysis, hence diminishing the clinical interpretive of their model. With results similar to our study, Wen et al. [41] presented BCCF-Net, which incorporates a channel-wise repeated fusion methodology. Their model achieved an AUC of 92.97% and an F1-score of 74.65% on PTB-XL. Although their channel modeling approach is unique, the comparatively low F1-score suggests inferior multi-label discriminating. Xia et al. [42] proposed 3D-STLNet employs 3D convolution and multi-head self-attention to capture spatial-temporal ECG features. Their model achieved an AUC of 92.5% and an F1-score of 80.43%, which is comparable, but not superior to our results. This indicates that although their spatial modeling is beneficial, it may lack substantial discriminative power in multi-label ECG scenarios. Although these studies report high performance, a significant portion of these studies employ data-splitting strategies that may lead to data leakage by allowing records from the same patient to appear in both the training and test sets. This situation can lead to an optimistic inflation of performance metrics through patient-dependent data leakage.

While many state-of-the-art deep learning architectures operate as black-box models with limited interpretability, the proposed CNN–FFT framework introduces a dual-domain structure that may enhance interpretability through explicit frequency-domain decomposition. By integrating a Fast Fourier Transform (FFT) branch prior to feature fusion, the model enables a structured representation of the input signal in both temporal and spectral domains, allowing for more comprehensive inspection of learned features. This dual-domain design provides a more transparent analysis of the frequency components contributing to the final prediction, as ECG signals are known to contain clinically relevant information distributed across frequency bands. In particular, rapid depolarization phenomena are associated with higher-frequency components, whereas slower repolarization-related processes are typically reflected in lower-frequency regions. By leveraging this decomposition, the proposed framework facilitates a more interpretable relationship between input signal characteristics and model outputs. Furthermore, this structured representation may support post-hoc interpretability by enabling the association of learned features with established cardiological intervals and morphological patterns, such as QRS complex duration and ST-segment changes, thereby improving the clinical traceability of model predictions and bridging the gap between data-driven learning and established pathophysiological understanding.

In this work, we presented a multi-label ECG classification model that combines CNN with handmade features (FFT) in a lightweight and computationally efficient manner. With a macro-AUC of 92.46%, an F1 score of 77%, and an exact match of 63.97% across all labels, our model produced competitive results and clinically more reliable performance. Additionally, our model demonstrated good calibration of the projected probabilities with a mean average precision (mAP) of 81.3% and a low calibration error (ECE) of 2.28%. This shows that the expected probability and the model’s actual confidence levels match precisely.

### Assessment of computing complexity

In order to successfully deploy tools in real-time clinical situations, efficiency and inference are very important. The model needs to find a balance between the computing demands and the accuracy of its predictions. This is because of the complexity of the classification process. Within the scope of this discussion, the utilization of CNN and FFT features guarantees that the model will continue to be computationally efficient. We used three essential metrics to evaluate the computational complexity of the model. These metrics are the number of trainable parameters, the floating-point operations per second (FLOPs), and the inference time. The model has roughly 86,853 parameters, which is a significantly lower number than other deep learning models that are considered to be state-of-the-art, for instance ResNet-50, which has 25.6 million parameters, and DenseNet, with 12.0 million parameters.

The FLOPs of the proposed model are estimated at 0.26 GFLOPs, which is considerably more efficient than robust algorithms such as DenseNet-121 and ResNet-50. determined based on an input size of (5000 × 12 × 1), with 5000 denotes the temporal dimension, 12 indicates a set of leads, and 1 signifies the dimension of the channel. The suggested model reaches a mean inference time of 1.19 ms per ECG signal with Batch Size of 32 (841.53 FPS) and 0.93 ms with Batch Size of 64 (1,078.81 FPS) running on Two NVIDIA Tesla V100-SXM2–16GB GPUs, it is appropriate for real-time deployment. The suggested model demonstrates an appropriate ratio regarding computational cost and classification accuracy when compared to baseline approaches. For example, although the number of parameters for ECA + SENet + ResNet [43] is 11.7 million and the FLOPs estimated at 10.56 GFLOPs, that of MVMS-Net is 0.39 million [17], and that of WildECG [40] is 0.313 million parameters significantly higher than that of our model (86,853 parameters). When compared with our method, the enhancement in multi-label classification efficacy (exact match rate) is negligible. This underscores the computational effectiveness of the suggested method, rendering it a viable option for implementation in resource-limited settings.

In addition to achieving competitive classification performance, the proposed CNN–FFT framework maintains a remarkably low computational footprint. As detailed in Table 8, our proposed model contains only 86,853 trainable parameters and requires 0.26 GFLOPs, making it substantially more compact than conventional deep learning architectures. Compared with robust baseline networks such as ResNet-50 (25.6 M parameters) and DenseNet-121 (12.0 M parameters), the proposed framework reduces the parameter count by more than 99% while maintaining competitive multi-label classification performance. Even when compared with existing lightweight architectures evaluated on the same dataset, such as MVMS-Net (0.39 M) and WildECG (0.313 M), our proposed hybrid approach remains approximately 3.5 to 4.5 times smaller, highlighting its suitability for deployment in resource-constrained edge devices and wearable healthcare systems.

Furthermore, in comparison to the existing approaches, our framework possesses a number of advantages. In particular, our approach takes into account the multi-label properties of the electrocardiogram (ECG) data, which makes it possible to detect multiple heart diseases at the same time. Likewise, with only 86,853 parameters and 0.26 GFLOPs, our model exhibits computational efficiency for real-time clinical and resource-constrained applications. This model can be used in demanding clinical environments where ECG signals need to be evaluated, whether on-site in hospital systems or via mobile remote monitoring solutions. The computational efficiency and optimized resource utilization of our method help reduce energy consumption during processing. Our approach is suitable for wearable ECG devices as well as real-time ECG analysis in the cloud, where processing resources may be limited.

Scalability is also important, as the models must be versatile for various clinical and portable applications with low processing requirements. The model’s moderate complexity facilitates its integration in hospital ECG monitoring systems that require real-time forecasts. Furthermore, the small number of parameters facilitate its deployment in portable and portable ECG monitoring systems, for instance Holter, enabling continual cardiac monitoring for patients at elevated risk. One of the main features of our model is its cloud suitability. With a frame rate of 1078.81 FPS, it is ideal for telemedicine. The proposed model has demonstrated potential for large-scale deployment in various healthcare settings by balancing low computational cost with fast inference time.

The well-calibrated nature of our model offers a crucial clinical advantage. Because poorly calibrated models can pose significant risks to clinical decision support systems by generating wrong predictions. The low ECE values observed in this study indicate that the model reliably classifies prediction probabilities and generates results suitable for risk-based clinical interpretation. Given that calibration analysis is largely absent from most published studies, this evaluation significantly strengthens the clinical relevance of our study, which is crucial in clinical or decision-making settings where probabilistic interpretation is paramount. Furthermore, the architecture’s low computational complexity improves its usability on resource-constrained hardware, unlike more complex frameworks. Thus, providing a viable alternative for field applications.

## Conclusion

In this work, we proposed a CNN-FFT hybrid architecture that integrates extracted time-domain features in the frequency-domain is proposed and systematically evaluated using the PTB-XL dataset. In the binary classification task, the proposed model can reliably distinguish between normal and abnormal ECG recordings, achieving an accuracy of 92.42% and an AUC value of 97.8%. Among the five-class multi-label classification, the model showed strong and balanced classification performance, especially in the NORM, MI, STTC, and CD classes. However, the high AUC values indicate that the model can capture discriminative hypertrophy-specific signaling components. This suggests that the decision-making capacity of the model can be maintained even in clinical scenarios with high similarity between classes. The calibration analysis of the model output showed low ECE value indicates that the model does not misleading predictions and exhibits appropriate behavior for risk-based clinical decision-making processes.

Ablation analysis results clearly demonstrate that FFT-based frequency features are the component providing the strongest contribution in the hybrid architecture in terms of both computational cost and discriminative power. It was observed that additional transformations such as Welch-PSD and DWT provided limited local improvements in some classes but did not increase overall performance and, in some cases, had a negative effect due to representational redundancy. Our finding supports the view that representing the fundamental spectral information in the ECG signal in a simple and physiologically meaningful way is more effective than complex multi-transformation strategies.

Overall, this framework is a balanced solution suitable for practical integration into clinical decision support systems. Thanks to its advantages such as high accuracy, robust calibration properties, interpretable spectral components, and relatively low computational cost. Future studies will evaluate multi-resolution spectral representations, signal quality-sensitive preprocessing steps, and advanced data augmentation strategies to improve performance, particularly in difficult-to-distinguish classes such as HYP. Furthermore, adapting the proposed architecture to low-power portable devices and real-time monitoring systems represents an important research direction that will further enhance the practical applicability of the method. Our findings further suggest that future ECG classification studies should prioritize reliability and proper evaluation protocols alongside performance improvements, particularly for clinical deployment scenarios.

## Supporting information

S1 Checklist TRIPOD+AI reporting checklist for prediction model development and evaluation. (PDF)

## References

[1] Tsao CW, Aday AW, Almarzooq ZI, Alonso A, Beaton AZ, Bittencourt MS, et al. Heart disease and stroke statistics-2022 update: A report from the American Heart Association. Circulation. 2022;145(8):e153–639. doi: 10.1161/cir.0000000000001052 35078371

[2] Roth GA, Forouzanfar MH, Moran AE, Barber R, Nguyen G, Feigin VL, et al. Demographic and epidemiologic drivers of global cardiovascular mortality. N Engl J Med. 2015;372(14):1333–41. doi: 10.1056/NEJMoa1406656 25830423 PMC4482354

[3] Kligfield P, Gettes LS, Bailey JJ, Childers R, Deal BJ, Hancock EW. Recommendations for the standardization and interpretation of the electrocardiogram: part I. J Am Coll Cardiol. 2007;49(10):1109–27. 17349896 10.1016/j.jacc.2007.01.024

[4] Maddox TM, Fry ET, Wilson BH. The cardiovascular workforce crisis: Navigating the present, planning for the future. J Am Coll Cardiol. 2024;83(3):466–9.doi: 10.1016/j.jacc.2023.12.001 38233020

[5] Kutluana G, Türker İ. Classification of cardiac disorders using weighted visibility graph features from ECG signals. Biomedical Signal Processing and Control. 2024;87:105420. doi: 10.1016/j.bspc.2023.105420

[6] Liu X, Wang H, Li Z, Qin L. Deep learning in ECG diagnosis: A review. Knowledge-Based Systems. 2021;227:107187. doi: 10.1016/j.knosys.2021.107187

[7] Rajpurkar P, Hannun AY, Haghpanahi M, Bourn C, Ng AY. Cardiologist-level arrhythmia detection with convolutional neural networks. 2017. doi: 170701836

[8] Hannun AY, Rajpurkar P, Haghpanahi M, Tison GH, Bourn C, Turakhia MP, et al. Cardiologist-level arrhythmia detection and classification in ambulatory electrocardiograms using a deep neural network. Nat Med. 2019;25(1):65–9. doi: 10.1038/s41591-018-0268-3 30617320 PMC6784839

[9] Clifford GD, Azuaje F. Advanced methods and tools for ECG data analysis. Artech House. 2006.

[10] Deo RC. Machine learning in medicine. Circulation. 2015;132(20):1920–30. doi: 10.1161/CIRCULATIONAHA.115.001593 26572668 PMC5831252

[11] Strodthoff N, Wagner P, Schaeffter T, Samek W. Deep learning for ECG analysis: Benchmarks and Insights from PTB-XL. IEEE J Biomed Health Inform. 2021;25(5):1519–28. doi: 10.1109/JBHI.2020.3022989 32903191

[12] Wang J, Qiao X, Liu C, Wang X, Liu Y, Yao L, et al. Automated ECG classification using a non-local convolutional block attention module. Comput Methods Programs Biomed. 2021;203:106006. doi: 10.1016/j.cmpb.2021.106006 33735660

[13] Safdar MF, Pałka P, Nowak RM, Faresi AA. A novel data augmentation approach for enhancement of ECG signal classification. Biomedical Signal Processing and Control. 2023;86:105114. doi: 10.1016/j.bspc.2023.105114

[14] Haque A, Akhtar N. Ensemble model for ECG Classification. In: 2022 5th International Conference on Multimedia, Signal Processing and Communication Technologies (IMPACT), 2022. 10.1109/impact55510.2022.10029230

[15] Martin H, Morar U, Izquierdo W, Cabrerizo M, Cabrera A, Adjouadi M. Real-time frequency-independent single-Lead and single-beat myocardial infarction detection. Artif Intell Med. 2021;121:102179. doi: 10.1016/j.artmed.2021.102179 34763801

[16] Vasconcelos L, Martinez BP, Kent M, Ansari S, Ghanbari H, Nenadic I. Multi-center atrial fibrillation electrocardiogram (ECG) classification using Fourier space convolutional neural networks (FD-CNN) and transfer learning. J Electrocardiol. 2023;81:201–6. doi: 10.1016/j.jelectrocard.2023.09.010 37778217

[17] Yang S, Lian C, Zeng Z, Xu B, Zang J, Zhang Z. A multi-view multi-scale neural network for multi-label ECG classification. IEEE Trans Emerg Top Comput Intell. 2023;7(3):648–60. doi: 10.1109/tetci.2023.3235374

[18] Yang F, Wang G, Luo C, Ding Z. Improving automatic detection of ECG abnormality with less manual annotations using siamese network. Annu Int Conf IEEE Eng Med Biol Soc. 2021;2021:1120–3. doi: 10.1109/EMBC46164.2021.9630333 34891484

[19] Zhu J, Lv J, Kong D. CNN-FWS: A model for the diagnosis of normal and abnormal ECG with feature adaptive. Entropy (Basel). 2022;24(4):471. doi: 10.3390/e24040471 35455133 PMC9025839

[20] Śmigiel S, Pałczyński K, Ledziński D. ECG signal classification using deep learning techniques based on the PTB-XL dataset. Entropy (Basel). 2021;23(9):1121. doi: 10.3390/e23091121 34573746 PMC8469424

[21] Śmigiel S, Pałczyński K, Ledziński D. Deep learning techniques in the classification of ECG signals using r-peak detection based on the PTB-XL dataset. Sensors (Basel). 2021;21(24):8174. doi: 10.3390/s21248174 34960267 PMC8705269

[22] Pałczyński K, Śmigiel S, Ledziński D, Bujnowski S. Study of the few-shot learning for ECG classification based on the PTB-XL dataset. Sensors (Basel). 2022;22(3):904. doi: 10.3390/s22030904 35161650 PMC8839938

[23] Elyamani HA, Salem MA, Melgani F, Yhiea NM. Deep residual 2D convolutional neural network for cardiovascular disease classification. Sci Rep. 2024;14(1):22040. doi: 10.1038/s41598-024-72382-3 39327440 PMC11427665

[24] Sharma K, Eskicioglu R. Deep learning-based ecg classification on raspberry pi using a tensorflow lite model based on ptb-xl dataset. 2022. doi: 220900989

[25] Li M, Chen W. FFT-based deep feature learning method for EEG classification. Biomedical Signal Processing and Control. 2021;66:102492. doi: 10.1016/j.bspc.2021.102492

[26] Gao Q, Omran AH, Baghersad Y, Mohammadi O, Alkhafaji MA, Al-Azzawi AKJ, et al. Electroencephalogram signal classification based on Fourier transform and Pattern Recognition Network for epilepsy diagnosis. Engineering Applications of Artificial Intelligence. 2023;123:106479. doi: 10.1016/j.engappai.2023.106479

[27] Fatimah B, Singhal A, Singh P. ECG arrhythmia detection in an inter-patient setting using Fourier decomposition and machine learning. Med Eng Phys. 2024;124:104102. doi: 10.1016/j.medengphy.2024.104102 38418030

[28] Lekkas G, Vrochidou E, Papakostas GA. Time–frequency transformations for enhanced biomedical signal classification with convolutional neural networks. BioMedInformatics. 2025;5(1):7. doi: 10.3390/biomedinformatics5010007

[29] Xiang J-Z, Wang Q-Y, Fang Z-B, Esquivel JA, Su Z-X. A multi-modal deep learning approach for stress detection using physiological signals: Integrating time and frequency domain features. Front Physiol. 2025;16:1584299. doi: 10.3389/fphys.2025.1584299 40236827 PMC11997569

[30] Wagner P, Strodthoff N, Bousseljot R-D, Kreiseler D, Lunze FI, Samek W, et al. PTB-XL, a large publicly available electrocardiography dataset. Sci Data. 2020;7(1):154. doi: 10.1038/s41597-020-0495-6 32451379 PMC7248071

[31] Zhou F, Chen L. Leadwise clustering multi-branch network for multi-label ECG classification. Med Eng Phys. 2024;130:104196. doi: 10.1016/j.medengphy.2024.104196 39160024

[32] Van Calster B, McLernon DJ, van Smeden M, Wynants L, Steyerberg EW, Topic Group ‘Evaluating diagnostic t, et al. Calibration: the Achilles heel of predictive analytics. BMC Med. 2019;17(1):230. doi: 10.1186/s12916-019-1466-7 31842878 PMC6912996

[33] Sörnmo L, Laguna P. Bioelectrical signal processing in cardiac and neurological applications. Academic Press. 2005.

[34] Clifford GD, Liu C, Moody B, Lehman LH, Silva I, Li Q, et al. AF classification from a short single lead ECG recording: The PhysioNet/computing in cardiology challenge 2017. In: 2017 computing in cardiology (CinC). IEEE. 2017. 10.22489/CinC.2017.065-469 PMC5978770 29862307

[35] Munõz A, Torres-Santamaria J, Iregui M, Romero E, Cruz-Roa A. Comparative Analysis of Deep Neural Network Architectures for Heart Disease Classification in Electrocardiography Signals. In: 2024 20th International Symposium on Medical Information Processing and Analysis (SIPAIM), 2024. 1–4. 10.1109/sipaim62974.2024.10783577

[36] Hancock EW, Deal BJ, Mirvis DM, Okin P, Kligfield P, Gettes LS. AHA/ACCF/HRS recommendations for the standardization and interpretation of the electrocardiogram: Part V: electrocardiogram changes associated with cardiac chamber hypertrophy. J Am Coll Cardiol. 2009;53(11):992–1002. doi: 10.1016/j.jacc.2008.12.015 19281932

[37] Surawicz B, Childers R, Deal BJ, Gettes LS. AHA/ACCF/HRS recommendations for the standardization and interpretation of the electrocardiogram: part III: Intraventricular conduction disturbances. J Am Coll Cardiol. 2009;53(11):976–81. doi: 10.1016/j.jacc.2008.12.013 19281930

[38] Selvam IJ, Madhavan M, Kumarasamy SK. Detection and classification of electrocardiography using hybrid deep learning models. Hellenic J Cardiol. 2025;81:75–84. doi: 10.1016/j.hjc.2024.08.011 39218394

[39] Yoo J, Jin Y, Ko B, Kim MS. K-labelsets method for multi-label ECG signal classification based on SE-ResNet. Appl Sci (Basel). 2021;11(16):7758.

[40] Avramidis K, Kunc D, Perz B, Adsul K, Feng T, Kazienko P, et al. Scaling representation learning from ubiquitous ECG with state-space models. IEEE J Biomed Health Inform. 2024;28(10):5877–89. doi: 10.1109/JBHI.2024.3416897 38935470

[41] Zeng W, Shan L, Chen Y, Ma Y, Du S. Interpretable deep learning framework for multi-label ECG classification: Enhancing cardiac diagnostics through feature attention and explainability. Biomedical Signal Processing and Control. 2026;112:108447. doi: 10.1016/j.bspc.2025.108447

[42] Wen W, Zhang H, Wang Z, Gao X, Wu P, Lin J, et al. Enhanced multi-label cardiology diagnosis with channel-wise recurrent fusion. Comput Biol Med. 2024;171:108210. doi: 10.1016/j.compbiomed.2024.108210 38417383

[43] Xia P, Zhang H, Yao Y, Xu L, Bai Z, Chen X, et al. Spatiotemporal 3-D variations modeling with self-attention for multilabel ECG classification. IEEE Sens J. 2024;24(11):18710–24.

