# Supplementary material for: A lightweight hybrid framework integrating convolutional neural networks and fast Fourier transform for reliable and calibrated ECG-based cardiac abnormality detection
Source: PLoS One. 2026 Jul 29;21(7):e0354834. doi: 10.1371/journal.pone.0354834 (PMC13419237; doi:10.1371/journal.pone.0354834)
Supplement: Attachment — Submitted filename: Response to Reviewers.docx [file pone.0354834.s003.docx]

**Response to Reviewers**

Manuscript ID: PONE-D-26-20240
Title: *A lightweight hybrid framework integrating convolutional neural networks and fast Fourier transform for reliable and calibrated ECG-based cardiac abnormality detection*

Dear Editor and Reviewers,

We sincerely thank the Academic Editor and the reviewers for their valuable time, constructive feedback, and positive assessment of our manuscript. We appreciate the time and effort invested in reviewing our work . We have carefully addressed all comments and revised the manuscript accordingly, and believe that the changes have improved the clarity and rigor of the study. All changes have been incorporated in the revised manuscript, with all new modifications and text additions highlighted in red for the convenience of the reviewers. Below, we provide a detailed respond to each comment point by point. Reviewer comments are reproduced in italics, followed by our responses.

**Response to the Academic Editor and Journal Requirements**

**1. PLOS ONE style requirements and file naming**

**Comment:** Please ensure that your manuscript meets PLOS ONE's style requirements, including those for file naming.

**Response:** We have carefully reviewed the manuscript and revised it to conform to PLOS ONE formatting requirements, including the title page, affiliations, manuscript structure, and file naming conventions. The revised submission includes:

- a clean manuscript file;
- a marked-up manuscript with tracked changes;
- and a separate response letter.

**2. Code sharing policy**

**Comment:** Please review PLOS ONE's guidelines on code sharing and ensure that your code is shared in a way that follows best practice and facilitates reproducibility and reuse.

**Response:** We have added a code availability in Data Availability statement and prepared the code for public release upon publication through a permanent public repository GitHub.

**3. Citation of recommended works**

**Comment:** If the reviewer comments include a recommendation to cite specific previously published works, please review and evaluate these publications.

**Response**: The reviewers did not recommend any specific publications for citation. However, during the revision process, we reviewed the relevant literature and incorporated additional references where appropriate to strengthen the manuscript and provide better contextualization of our work.

**4. Reference list accuracy and retracted papers**

**Comment:** Please review your reference list to ensure that it is complete and correct.

**Response:** We carefully checked the reference list, corrected any inconsistencies, and verified the status of all cited works. Where relevant, we either removed problematic references in the manuscript.

**Response to Reviewer #1**

We thank Reviewer #1 for the positive assessment of our study and for the helpful suggestions.

***Reviewer #1****: In this work, Malik and Aras propose a hybrid machine learning architecture based on convolutional neural networks and fast Fourier transform features to extract relevant information from ECG signals in a public dataset. They show that this approach achieves strong performance in classifying normal versus abnormal ECG recordings while using a relatively small number of model parameters. The results also suggest that Fourier-based features substantially improve model performance. Overall, this is an interesting study with comprehensive analysis and informative results that may be useful for the biomedical signal processing community. However, several points should be addressed to improve the quality and clarity of the work.*

***Comment 1****: The definition of "sick" subjects in line 189 requires clarification, as the current wording is too general.*

**Response:** Thank you for this important note. We agree that the term "sick" was too general and lacked clinical precision. In the revised manuscript, we have replaced this terminology with "subjects with cardiac abnormalities". Furthermore, we have explicitly clarified that this category encompasses all patients diagnosed with at least one of the four pathological diagnostic super classes present in the PTB-XL dataset (MI, STTC, HYP, and CD) in Dataset subsection. (see Page 9 and 11)

***Comment 2****: The authors should discuss recent relevant studies that use Fourier decomposition for feature extraction and machine learning for biomedical signal analysis. This would better position the contribution of the present study relative to prior work.*

**Response:** Thank you for this constructive recommendation. We agree that contextualizing our work with recent literature on Fourier-based feature extraction in biomedical signal processing strengthens the manuscript. In the revised version, we have added a dedicated paragraph in the Introduction section discussing recent studies that leverage Fourier decomposition alongside machine learning and deep learning for physiological signals. These additions highlight the growing interest in combining spectral-domain information with machine learning models and further justify the integration of FFT-derived features within the proposed CNN-FFT framework. (see Page 6-7)

***Comment 3****: In the population categorization, the authors should report the age distribution across the five categories of healthy individuals and individuals with conditions. It would also be helpful to assess whether there are statistically significant differences in age or BMI among these groups.*

**Response:** Thank you for this excellent suggestion, which substantially enhances the clinical and demographic characterization of the dataset. To address this comment, we extracted the baseline population metadata from the PTB-XL database. We have included a new table (Table 2) in the revised manuscript reporting the precise sample size (N) , Median and Interquartile Range [IQR] of the age distribution across the five diagnostic groups. A non-parametric Kruskal-Wallis test was performed to evaluate the statistical differences, demonstrating a highly significant age variation among the cohorts (p < 0.001). Pathological groups (MI, STTC, and CD) expectedly display a significantly older demographic baseline compared to normal healthy controls. Regarding BMI, we systematically reviewed the clinical metadata and noted that anthropometric variables (height and weight) contain a very high rate of missing values in the publicly available PTB-XL database. To avoid introducing sampling bias or relying on unreliable large-scale data imputation, we have restricted our clinical baseline analysis strictly to the verified age parameters. (see Page 12)

***Comment 4****: One major advantage of the proposed approach is the reduced number of trainable parameters. Although the authors briefly discuss this point in the Discussion section, it would be valuable to highlight it more explicitly, preferably by summarizing the comparison in a table.*

**Response**: We thank the reviewer for this valuable suggestion. We completely agree that the lightweight nature of the proposed architecture constitutes an important contribution that deserves more explicit visibility. To address this and avoid representational redundancy, we have revised the "Assessment of computing complexity" subsection and expanded the discussion surrounding our comparative table 8. Specifically, the title of Table has been updated to explicitly reflect both predictive performance and trainable parameters. Furthermore, we have incorporated dedicated paragraphs to highlight the substantial reduction in architectural footprint achieved by our framework compared with both heavy conventional backbones and existing lightweight state-of-the-art models. Additional discussion has been incorporated into Section 4 (Discussion) under the “Assessment of computing complexity” subsection. (see Page 29 and 31)

***Comment 5:*** *The authors should better explain why distinguishing the HYP class was the most challenging task from a clinical perspective.*

**Response:** We thank the reviewer for this insightful clinical comment. We agree that expanding the discussion of the HYP class strengthens the clinical relevance of the manuscript. From a clinical perspective, hypertrophy-related ECG patterns are often subtle and heterogeneous, and they may overlap with ST/T segment abnormalities and conduction disturbances. This overlap makes the HYP class inherently more difficult to distinguish than the other diagnostic categories, both for automated models and in routine clinical interpretation. In the revised manuscript, we expanded the Discussion section to clarify these electrophysiological overlaps and to explain why the decision boundaries for HYP are particularly ambiguous. We have revised and expanded the paragraph in Section 4 (Discussion), under the "Benchmarking and performance evaluation" subsection. (see Page 25-26)

**Response to Reviewer #2**

We thank Reviewer #2 for the constructive and detailed feedback. The comments were very helpful in improving the manuscript.

***Reviewer #2****: The authors develop machine learning models for binary and multi-class classification of ECG arrhythmia using the PTB-XL dataset. The models appear to be lightweight and perform well at the task, and the methods are well-described, however, require some clarifications for the reader. For this reason, I recommend minor revisions to make the following clarifications ahead of publication.*

***Comment 1****: Reporting Guidelines
As the authors are building a clinical diagnostic model, the authors should complete the appropriate reporting checklist from the EQUATOR network and include a statement regarding which checklist has been utilized as well as make the minor revisions to the manuscript to comply with the appropriate reporting guidelines. I believe the TRIPOD-AI checklist may be most relevant here, but I may be slightly mistaken. Furthermore, within the manuscript I have found no statement on the availability of the models built.*

**Response:** We thank the reviewer for this important recommendation. To improve reporting transparency and reproducibility, we reviewed the manuscript with reference to the TRIPOD-AI reporting guideline and revised the manuscript accordingly where appropriate. A completed TRIPOD-AI checklist is provided as supplementary material. In addition, the source code will be made publicly available upon acceptance of the manuscript in accordance with PLOS ONE's open science and reproducibility requirements. (see Page 10 and 34)

***Comment 2****: Training Epochs
Were the models trained in a single epoch on the first (1-8) folds of the PTB-XL dataset? Or were they allowed to view the training data for multiple passes? If so, how many epochs and how was the number determined?*

**Response**: We thank the reviewer for this important question. The models were trained over multiple epochs rather than a single pass through the training data. During hyperparameter optimization, the number of training epochs was explored within the range of 10–50 epochs, and the best-performing configurations without introducing performance degradation or overfitting were obtained using maximum epoch values of 20 and 40 epochs for the binary and multi-class tasks, respectively. The manuscript has been revised to explicitly clarify the training procedure. (see Page 17)

***Comment 3****: Confusion Matrix Thresholds
Could the authors make clear how thresholds were set for the confusion matrices depicted throughout the manuscript? (e.g., Fig. 3, Fig. 5). Both the method and the subset of the training / validation dataset used in determining the thresholds.*

**Response**: We thank the reviewer for highlighting this important methodological detail. For both the binary and multi-label classification tasks, a standard decision threshold of 0.5 was applied to the output probabilities generated by the Sigmoid activation function. In the multi-label setting, each diagnostic category was evaluated independently, and labels with predicted probabilities greater than or equal to 0.5 were assigned as positive. The threshold was applied exclusively to predictions obtained from the independent test set (fold 10) during final model evaluation. We have revised the manuscript to explicitly clarify the thresholding procedure used to generate the reported confusion matrices*.* (see Page 17)

***Comment 4****: Confidence Intervals
Could the authors clarify how the confidence intervals in Table 4 are computed?*

**Response:** We thank the reviewer for pointing out this omission. We have clarified that the reported 95% confidence intervals were estimated using bootstrap resampling. We have added a sentence in Section 2 under the "Model training and evaluation metrics" subsection to explicitly state that a non-parametric bootstrap resampling approach with 1,000 iterations was used to compute the 95% confidence intervals. (see Page 17)

***Comment 5****: Ablation Study
It is unclear whether the addition of FFT information represents a meaningful improvement in model performance or is simply overfitting in the presence of additional variables. Some statistical comparison of the improvement that is robust to the confidence intervals / model stability may support this studies’ assertions better (e.g., DeLong test for the comparison)*

**Response**: We thank the reviewer for this important observation. The objective of the ablation study was to evaluate the contribution of frequency-domain representations to the proposed framework. As shown in Table 6, the integration of FFT-derived features yielded the highest performance among the evaluated configurations.

We acknowledge that the FFT transformation does not by itself establish the statistical significance of the observed improvement. However, it is important to note that the FFT branch operates as a deterministic, non-trainable transformation of the input signal and therefore does not introduce additional trainable parameters within the feature extraction stage. Rather than increasing model complexity through additional learned filters, the FFT provides complementary frequency-domain information that is fused with the temporal representations extracted by the CNN. Furthermore, the FFT branch acts as a structured inductive bias that enables the model to exploit clinically relevant spectral characteristics of ECG signals, resulting in consistent performance improvements across both the binary and multi-label classification tasks. We have clarified this point in the revised manuscript and moderated our discussion to avoid overstating the contribution of the FFT component. (see Page 21-22)

***Comment 6****: Interpretability
Within the discussion section the authors consistently mention that other designs lack interpretability – however, they do not discuss approaches to interpretability of their model in depth. Additional context on this could be provided within this section.*

**Response**: We thank the reviewer for this constructive feedback. We agree that providing a deeper discussion on the interpretability of our hybrid CNN–FFT model strengthens our claims. We have revised the Discussion section to provide a more detailed explanation of the interpretability of our framework. Specifically, we now clarify how the explicit frequency-domain decomposition introduced by the FFT branch enables a structured representation of ECG signals across temporal and spectral domains, improving the interpretability of learned features. We also highlight the clinical relevance of frequency-band information in relation to established cardiological patterns such as QRS complexes and ST-segment variations. In the revised manuscript, we have added a dedicated paragraph on model interpretability in Section 4 (Discussion). (see Page 28-29)

***Comment 7****: Line 439, fix typographical error: “the The”.*

**Response**: We thank the reviewer for carefully reading the manuscript. The typographical error identified by the reviewer has been corrected.

We believe that these revisions have significantly improved the clarity, technical accuracy, and presentation of the manuscript. We sincerely hope that the revised version meets the journal’s requirements and is suitable for publication.

Thank you again for your time and consideration.

Kind regards

Abdoul Malik

(On behalf of all authors)
